# Trends in public attitudes to permitting cannabis for recreational use: analysis of Irish survey data since 2002

**DOI:** 10.1093/eurpub/ckad054

**Published:** 2023-04-08

**Authors:** Deirdre Mongan, Seán R Millar, Claire O’Dwyer, Brian Galvin, Bobby P Smyth

**Affiliations:** Health Research Board, Dublin 2, Ireland; Health Research Board, Dublin 2, Ireland; School of Public Health, University College Cork, Cork, Ireland; School of Psychology, University College Dublin, Belfield, Dublin 4, Ireland; Health Research Board, Dublin 2, Ireland; Department of Public Health & Primary Care, Trinity College Dublin, Dublin, Ireland

## Abstract

**Background:**

There has been considerable debate around the liberalization of cannabis laws in many countries. Given recent changes in cannabis policy, and the current discussion regarding cannabis legalization in Ireland, the aim of this study was to examine changes in attitudes over time towards permitting recreational cannabis use.

**Methods:**

We analyzed data from Ireland’s 2002/03 (*n* = 4918); 2006/07 (*n* = 4967); 2010/11 (*n* = 5119); 2014/15 (*n* = 5937); 2019/20 (*n* = 3982) National Drug Prevalence Surveys. Multivariable logistic regression analyses were used to examine factors associated with being in favour of the use of cannabis for recreational purposes.

**Results:**

The results indicate that there is minority support for permitting recreational cannabis use, which ranged from 19.1% in 2006/07 to 29.9% in 2019/20. In multivariable analysis being male and living in Dublin were significant predictors of agreeing with recreational cannabis use, as were being either a recent or past cannabis user, knowing cannabis users, perceiving cannabis use as not being a great risk, and not disapproving of cannabis use. Subjects aged less than 35 years and those who had completed primary education only were significantly less likely to agree with permitting recreational cannabis use.

**Conclusion:**

The results from this study indicate that there is minority support for allowing recreational cannabis use. Support was highest among recent cannabis users, consistent with previous studies. The relative lack of support for recreational cannabis use among younger respondents was surprising and warrants further research.

## Introduction

Cannabis was declared an illicit drug in 1961 in countries that signed the United Nations Single Convention on Narcotic Drugs, where it was classified as a drug that can lead to abuse and ill effects and that has no medical use.^1^ However, in recent years there has been a well-organized international movement promoting the legalization of cannabis^2^ and a number of jurisdictions throughout the world have legalized the recreational use of cannabis. In the USA, the states of Colorado and Washington legalized the sale and production of cannabis for recreational use in 2012, with the first sales occurring in 2014.^3^ As of November 2022, a further 19 states in the USA have enacted legislation to regulate cannabis for recreational use.^4^ In Canada, the sale of cannabis for recreational use became legal in 2018, with the declared aim of protecting public health and safety.^5^ Uruguay introduced restrictive laws permitting recreational cannabis use in 2013 with the goal of removing organized crime from the cannabis supply and protecting public health and safety.^6^ In the European Union, no countries have permitted any legal sale for recreational purposes, with the exception of the Dutch system of cannabis sale through coffeeshops.^7^ However, a new coalition government in Germany agreed in late 2021 to regulate the sale of cannabis to adults for recreational purposes, while Luxembourg has also announced that it will legalize the production, sale, and consumption of cannabis.^8^ In contrast, a national referendum held in New Zealand in 2020 to legalize the sale and supply of cannabis for recreational use failed.^9^

There has been much debate around the liberalization of cannabis laws in Ireland. A campaign calling for the decriminalization of all drug use commenced in 2012.^10^ This was expanded and built upon by other non-governmental organizations with the assistance of funding from donors of the international cannabis legalization movement.^11^ There has also been a very visible campaign for medical cannabis since 2014. This campaign has been supported and sponsored by a small number of very wealthy cannabis legalization activists and by the emerging North American cannabis industry. Legislation was passed in 2019 to allow for a Medical Cannabis Access Programme to come into operation in Ireland on a pilot basis for 5 years. To date, there have been no changes to the legal status of cannabis for recreational use in Ireland. However, in February 2023, the Irish Government announced its intention to establish a Citizens’ Assembly on Drug Use to consider the legislative and policy changes that may reduce the harmful impacts of illicit drugs.^12^

Analysis of US survey data suggests that support for cannabis legalization is higher among men, younger people, those who have ever used cannabis, and those who do not perceive cannabis use to be risky.^13^ However, in New Zealand, demographic variables were not found to be predictors of support to legalize recreational cannabis use. Instead, perceptions of the health risk and moral views of cannabis use, agreeing with the use of cannabis for medical reasons and living in a small town were associated with support^14^ In Australia, support for recreational cannabis legalization was related to high income, past or recent cannabis use, high-risk drinking and current tobacco use.^15^

Public support can influence, and be influenced by, national cannabis policy. In the USA, where most research on cannabis attitudes has been undertaken, support for cannabis legalization began to grow through the mid-1970s, fell in the 1980s and began to increase again after the early 1990s. Repeated cross-sectional data from the US General Social Survey show that support for legalization was just 16% in 1990 but increased to 30% in 2000, 44% in 2010 and 57% in 2016, with similar trends observed in Gallup polls in the USA.^16–18^ Perceptions of the risks associated with cannabis use have also decreased over time both in Ireland and elsewhere. Among Irish adults aged 15–64 years, perceived great risk from regular cannabis use increased from 57% in 2002/03 to 64% in 2010/11 before decreasing to 52% in 2019/20.^19^ European schoolchildren reported a decrease in perceived great risk from regular cannabis use from 71% in 2007 to 59% in 2019.^20^ Similar trends have been reported in the USA.^21^^,^^22^ However, in the USA, these trends have been accompanied by an increase in adult cannabis use^23^ and adverse cannabis consequences, including cannabis use disorders, fatal crashes and emergency department visits.^24^ In addition, rates of cannabis use among USA and Canadian youth are 2–3 times higher than those in Europe.^25^

In Ireland, there has also been an increase in cannabis use and cannabis-related harm that has accompanied by a decrease in risk perception due to cannabis use.^19^^,^^26^ It has been noted that public opinion has played a major role in cannabis liberalization in the USA. Given recent changes in cannabis policy, and the current debate regarding cannabis legalization in Ireland, understanding trends in public opinion and the characteristics of supporters may help inform policy around cannabis regulation. Therefore, the aim of this study was to examine changes in attitudes towards recreational cannabis use over time using data from five large nationally representative surveys. In particular, we explored characteristics associated with public attitudes towards the use of cannabis for recreational purposes.

## Methods

### Data source

We analyzed data from Ireland’s National Drug Prevalence Surveys; to date, this survey has been conducted five times: 2002/03, 2006/07, 2010/11, 2014/15 and 2019/20. These cross-sectional surveys collect information on alcohol and tobacco consumption and drug use among the general population in Ireland. They also survey attitudes and perceptions relating to tobacco, alcohol, and other drug use and record the impact of drug use on communities. The surveys recruited stratified, clustered samples of people living in private households in Ireland. For the first three surveys, adults aged 15–64 years were recruited, but this was changed in the 2014/15 survey to include adults over 64 years of age. The number of respondents aged 15–64 years in each survey was 4918 in 2002/03; 4967 in 2006/07; 5119 in 2010/11; 5937 in 2014/15; and 3982 in 2019/20. The sample size in 2019/20 was lower than previous surveys as data collection had to be prematurely ended due to the introduction of restrictions on movement arising from the coronavirus disease 2019 pandemic.

### Study design

Each survey was undertaken using the same methodology. The sampling frame used was the GeoDirectory; this is a list of all addresses in the Republic of Ireland, and it distinguishes between residential and commercial establishments. A three-stage process was used to construct the sample for each survey. The first stage involved stratifying the population into 10 former health board regions in Ireland. In the second stage of stratification, electoral divisions were selected as the primary sampling units across the 10 regions. Before selection, the primary sampling units were ranked by the following socio-demographic indicators: population density, male unemployment and social class, to ensure that a representative cross-section of areas was included. Finally, in each primary sampling unit, addresses were chosen randomly, and at each address, one person was randomly selected to participate in the survey. For each survey, the achieved sample was weighted by sex, age and region to maximize its representativeness of the general population. The survey involved a face-to-face interview in the participant’s home. The home interviews were conducted by trained interviewers using Computer Assisted Personal Interviewing. The response rates for the surveys ranged from 61% to 70%. No data on non-respondents were collected. Ethical approval to conduct the surveys was granted by the Royal College of Physicians in Ireland, and all participants gave written informed consent. A more comprehensive description of the surveys’ methodology has been detailed elsewhere.^27^

### Attitudes towards permitting cannabis use

Respondents were asked about the extent to which they agreed with the following statement: ‘People should be permitted to take cannabis for recreational reasons’. Response categories were presented on a 6-point Likert scale (‘fully agree’, ‘somewhat agree’, ‘neither agree or disagree’, ‘somewhat disagree’, ‘fully disagree’ and ‘don’t know’). These responses were collapsed into three categories: ‘agree’ (derived from ‘fully agree’ and ‘somewhat agree’); ‘neutral’ (derived from ‘neither agree or disagree’ and ‘don’t know’); and ‘disagree’ (derived from ‘somewhat disagree’ and ‘fully disagree’).

### Personal characteristics

Socio-demographic variables for this study included sex, age, marital status, educational attainment, employment, region of residence and survey year. In relation to age, as the first three surveys only included respondents aged 15–64 years the analysis presented here are confined to that age group only. Cannabis use status was classified into ‘never user’ (those who had never used cannabis), ‘past user’ (those who had used cannabis, but not in the last year) and ‘recent user’ (those who had used cannabis in the last year). All respondents were asked if they knew somebody who used cannabis. Perceived acceptability of cannabis was assessed by asking adult respondents how they felt about people smoking cannabis occasionally (‘do not disapprove’, ‘disapprove’ and ‘disapprove strongly’). We dichotomized this variable into ‘do not disapprove’ vs. ‘disapprove and disapprove strongly’. Perceived risk of cannabis use was assessed by asking respondents about the risk of regularly smoking cannabis (‘no risk’, ‘slight risk’, ‘moderate risk’ and ‘great risk’). We dichotomized this variable into those reporting ‘great risk’ vs. all others.

### Statistical analysis

The five datasets were coded and merged. Cross-tabulations were used to compare the distribution of agreement with recreational cannabis use across socio-demographics and cannabis-related variables. Multivariable logistic regression analyses were performed to estimate adjusted odds ratios of agreeing with or being neutral towards recreational cannabis use. The ability of variables identified in multivariable analysis to separate those who agreed with or were neutral towards cannabis use from those who disagreed was evaluated using the *c* statistic. For all analyses, a *P*-value of less than 0.05 was considered to indicate statistical significance. There were low levels of missing data. Missing independent variable data were thus assumed to be ignorable and missing at random. Data were analyzed using Stata Version 15.1 (Stata Corporation, College Station, TX, USA). Results are displayed using weighted data.

## Results

**Figure 1:**
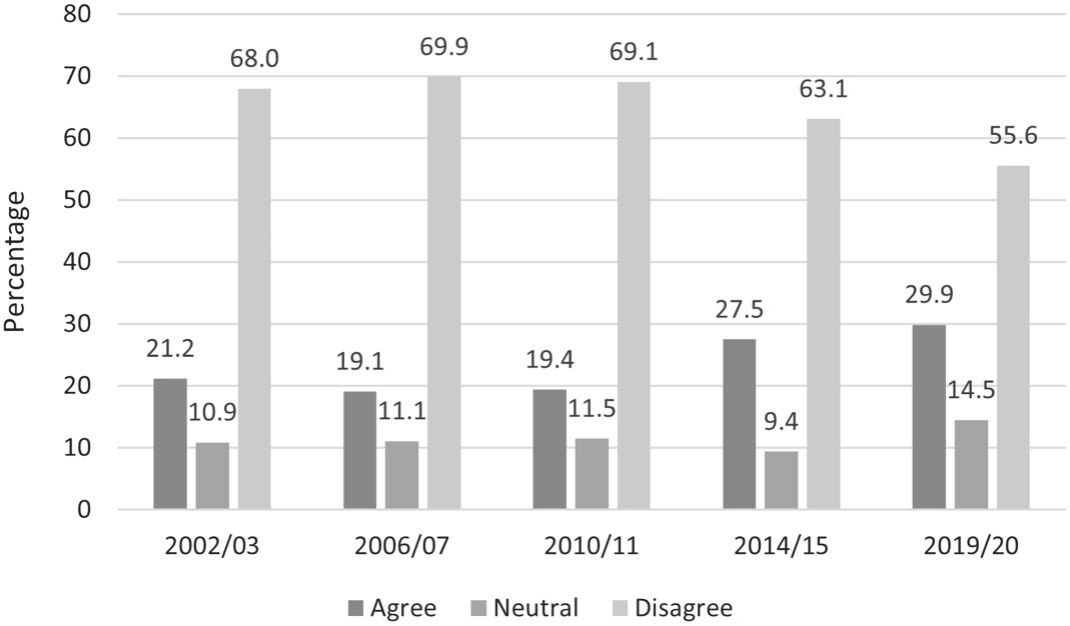
Level of agreement with recreational cannabis use among 15- to 64-year-old 2002/03–2019/20

Overall, agreement for permitting cannabis for recreational use among 15- to 64-year-olds was low across the five surveys; however, it has increased from 19.1% in 2006/07 to 29.9% in 2019/20 (figure 1). There was a decrease in disagreement for recreational use (from 68.0% in 2002/03 to 55.6% in 2019/20). In 2019/20, when respondents of all ages are included, 26.2% agreed, 13.6% were neutral and 60.2% disagreed with permitting recreational cannabis use; in 2014/15 27.5% agreed, 9.5% were neutral and 63.0% disagreed.

For each survey year, recent and past cannabis users were more likely to agree with recreational use compared with never users. In 2019/20, agreement among never users was 18.2%, compared with 59.3% for past users and 82.8% for recent users (table 1). Agreement with recreational cannabis use among past and recent cannabis users decreased in 2006/07 and 2010/11 before increasing in 2014/15 and 2019/20. In each survey, recent users aged 15–24 years were less likely than older cohorts to agree with recreational use.

**Table 1:** Proportion of respondents who agree with permitting recreational cannabis use; cannabis use history by age group (%)

	2002/03	2006/07	2010/11	2014/15	2019/20
Never users					
All	12.8	10.6	11.0	15.8	18.2
15–24	12.8	8.4	9.7	18.5	20.7
25–34	17.5	12.1	11.1	16.5	20.9
35+	11.0	10.8	11.4	14.7	16.7
*P*-value	<0.001	0.092	0.606	0.104	0.106
Past users					
All	53.2	39.5	37.8	48.9	59.3
15–24	53.6	34.6	24.1	45.6	61.8
25–34	48.4	36.1	35.0	47.7	66.0
35+	57.2	44.1	43.6	50.0	57.0
*P*-value	0.226	0.114	0.001	0.278	0.350
Recent users					
All	80.7	70.7	65.6	78.0	82.8
15–24	80.6	63.5	56.1	73.4	75.2
25–34	75.2	79.5	68.5	81.1	83.6
35+	89.3	73.2	76.8	80.9	93.4

Agreement with recreational cannabis use was higher among males, 25- to 34-year-olds, single respondents, unemployed respondents, those still in education and respondents living in Dublin. Agreement was also higher among recent cannabis users, those who knew cannabis users, those who did not perceive great risk associated with cannabis, and those who did not disapprove of cannabis use (table 2).

**Table 2:** Distribution of attitudes towards recreational use by socio-demographic and cannabis variables (%)

	Total (*n* = 25 553)	Agree (*n* = 6007)	Neutral (*n* = 2899)	Disagree (*n* = 16 647)
Year				
2002/03	19.2	21.2	10.9	68.0
2006/07	19.4	19.1	11.1	69.9
2010/11	20.0	19.4	11.5	69.1
2014/15	23.1	27.5	9.4	63.1
2019/20	18.4	29.9	14.5	55.6
Sex				
Male	50.0	30.1	11.9	58.0
Female	50.0	17.0	10.8	72.3
Age group				
15–24	20.5	24.8	13.3	62.0
25–34	23.6	28.7	13.3	58.0
35–64	55.9	20.9	9.8	69.3
Marital status				
Single/never married	37.5	27.3	13.3	59.4
Married/cohabiting	56.8	21.0	10.1	68.8
Divorced/separated/widowed	5.7	22.9	10.4	66.7
Education				
Primary/none	6.0	16.2	7.9	75.9
Completed secondary	28.8	21.8	9.8	68.4
Completed third level	43.6	24.8	12.1	63.2
Still in education	21.6	25.0	12.7	62.3
Employment				
Employed	61.9	25.0	11.3	63.7
Unemployed	10.9	28.4	11.8	59.8
Student	13.0	23.9	13.4	62.8
Other	14.2	13.2	9.0	77.8
Region				
Dublin	28.5	30.2	12.3	57.4
Rest of Ireland	71.5	20.8	11.0	68.2
Cannabis use				
Never user	76.4	13.7	11.1	75.3
Past user	17.1	47.2	13.6	39.2
Recent user	6.5	75.7	8.8	15.5
Know cannabis users				
No	56.4	11.3	10.2	78.5
Yes	43.6	39.3	12.7	48.0
Risk perception				
Great risk	59.5	10.0	7.7	82.3
Not a great risk	40.5	43.4	16.0	40.6
Social acceptability				
Disapprove	71.7	8.3	9.1	82.6
Do not disapprove	28.3	62.3	14.9	22.9

Table 3 presents multivariable associations between socio-demographic and cannabis variables and the likelihood of agreeing with or being neutral towards cannabis for recreational use. Being male and living in Dublin were significant predictors of agreeing with recreational cannabis use, as were being either a recent or past cannabis user, knowing cannabis users, perceiving cannabis use as not being a great risk, and not disapproving of cannabis use. Subjects aged less than 35 years and those who had completed primary education only were significantly less likely to agree with permitting recreational cannabis use. The year that the survey took place was also significantly related to agreeing with recreational use. The *c* statistic for this multivariable model was 0.90 (95% CI 0.89–0.90), indicating that the model had excellent ability at discriminating cases who agreed with permitting cannabis for recreational use from those who disagreed.

**Table 3:** Adjusted odds ratios of agreeing with permitting cannabis for recreational use (the response ‘disagree’ is the reference category)

	Neutral vs. disagree	Agree vs. disagree
	OR (95% CI)	OR (95% CI)
Year		
2019/20	1 (reference)	1 (reference)
2014/15	0.55 (0.46–0.67)	0.85 (0.72–1.01)
2010/11	0.62 (0.52–0.74)	0.49 (0.42–0.59)
2006/07	0.52 (0.44–0.62)	0.40 (0.34–0.47)
2002/03	0.58 (0.49–0.69)	0.53 (0.45–0.62)
Sex		
Female	1 (reference)	1 (reference)
Male	1.21 (1.08–1.35)	1.66 (1.50–1.85)
Age group		
35–64	1 (reference)	1 (reference)
25–34	1.09 (0.95–1.25)	0.79 (0.69–0.91)
15–24	0.87 (0.71–1.08)	0.55 (0.44–0.69)
Marital status		
Single/never married	1 (reference)	1 (reference)
Married/cohabiting	0.80 (0.70–0.93)	0.89 (0.78–1.02)
Divorced/separated/widowed	0.96 (0.77–1.21)	0.94 (0.76–1.15)
Education		
Completed third level	1 (reference)	1 (reference)
Primary/none	0.63 (0.48–0.82)	0.70 (0.54–0.89)
Completed secondary	0.82 (0.72–0.94)	0.97 (0.85–1.10)
Still in education	0.93 (0.77–1.12)	0.98 (0.82–1.17)
Employment		
Employed	1 (reference)	1 (reference)
Unemployed	1.14 (0.94–1.37)	1.09 (0.92–1.31)
Student	1.12 (0.85–1.46)	1.04 (0.79–1.36)
Other	0.92 (0.78–1.07)	0.93 (0.79–1.09)
Region		
Rest of Ireland	1 (reference)	1 (reference)
Dublin	1.08 (0.96–1.23)	1.20 (1.06–1.35)
Cannabis use		
Never user	1 (reference)	1 (reference)
Past user	1.24 (1.06–1.45)	1.93 (1.70–2.21)
Recent user	1.22 (0.90–1.66)	4.21 (3.31–5.35)
Know cannabis users		
No	1 (reference)	1 (reference)
Yes	1.18 (1.04–1.33)	1.77 (1.58–1.99)
Risk perception		
Great risk	1 (reference)	1 (reference)
Not a great risk	2.78 (2.48–3.11)	3.29 (2.96–3.67)
Social acceptability		
Disapprove	1 (reference)	1 (reference)
Do not disapprove	3.82 (3.37–4.35)	11.89 (10.65–13.27)

Notes: Odds ratios are adjusted for all variables in the table. Odds ratios and 95% CIs are rounded to two decimal places.

The significant predictors of being neutral towards recreational cannabis use were being male, past use of cannabis, knowing cannabis users, not perceiving great risk and not disapproving of cannabis use. Married respondents and those with primary or secondary education only were less likely to be neutral towards cannabis use. The *c* statistic for this multivariable model was 0.75 (95% CI: 0.73–0.76), indicating that the model had a moderate ability at discriminating cases who agreed with permitting cannabis for recreational use from those who were neutral.

## Discussion

The public and political interest in legalizing cannabis use has increased considerably in recent years. The results from this study indicate that support for permitting recreational cannabis use in Ireland is low at 29.9%, but there has been an increase in support since 2010/11. The reasons behind this increase in support are not clear, but the recent, predominantly positive, public discourse in Ireland in relation to the potential medical utility of cannabis-based products may have led to Irish people having a more positive view of cannabis in general.^28^ Nevertheless, this level of support is much lower than in the USA, where support for legalizing cannabis for recreational use was 60% in 2015^29^ and lower than Australia, where support was 40% in 2016.^17^

The trends in support for permitting recreational use in Ireland since 2002/03 are non-linear and are difficult to explain. Compared to other jurisdictions that have seen more linear increases in support of recreational cannabis use,^18^ support in Ireland decreased significantly between 2002/03 and 2010/11 before significantly increasing to the highest observed level of support in 2019/20. Given the fact that the exact same methodology was used to conduct each survey, we are confident that this trend is not due to survey design and that some other factors must be at play. The decrease in support observed between 2002/03 and 2006/07 is also surprising as cannabis use in Ireland actually increased between these two time points;^19^ several studies have reported that support for permitting cannabis use is positively correlated with use.^30^^,^^31^ The more negative attitudes towards cannabis over the period from 2003 to 2011 may possibly be related to other changes in cannabis use and related harms in Ireland. Firstly, the type of cannabis being used changed from relatively low potency resin (‘hash’) to higher potency herbal cannabis (‘weed’). The increase in use and move towards higher potency products in Ireland has coincided with a substantial increase in cannabis addiction and cannabis-related psychiatric problems.^26^

Our multivariable models found that sex, age, education and region of residence were significant predictors of supporting recreational cannabis use. This differs to research from New Zealand which found that support for recreational cannabis legalization was not associated with socio-demographic variables.^14^ In the USA, where support for cannabis legalization has grown, there is no evidence that the changes within any particular socio-demographic group have been responsible for the overall change in attitude.^17^ The relative lack of support for recreational cannabis among younger respondents in this study was surprising as younger people in Ireland are more likely to use cannabis compared with older adults.^19^ It is possible that younger users may be more aware of the potential harmful effects of cannabis given the elevated rates of use amongst their peers and therefore less likely to agree with its legalization. In each of the five surveys, recent users aged 15–24 years were less likely than older recent users to agree with recreational cannabis use. The explanation as to why younger people in Ireland, especially recent users aged 15–24 years, are most opposed to permitting cannabis use warrants further study.

The strongest predictors for supporting recreational cannabis use were not disapproving of cannabis use, being a recent user and not perceiving cannabis as being a great risk. This is consistent with international research; in New Zealand, support for legalizing recreational cannabis use was associated with believing it was morally acceptable to use cannabis while considering cannabis use to be a high health risk was a predictor of not supporting the legislation.^14^ In the Netherlands, 7% of cannabis users and 50% of non-users were in favour of prohibiting cannabis,^32^ while research from Australia reported that being a cannabis user had a large positive effect on support for legalization.^17^^,^^31^ While we observed that being a past cannabis user also had a positive effect on support for legalization, the magnitude of association was less than half the size of the effect of being a recent cannabis user. Overall, it is interesting that in 2019/20 40.7% of past users and 17.2% of recent users were either neutral or disagreed with permitting a behaviour in which they had engaged. Given their lived experience of use, it would be helpful to obtain a greater understanding of the rationale for disagreement amongst such groups.

To date, there has been little political appetite to liberalize the Irish legislation regarding recreational cannabis use, and the results presented here would suggest a reluctance among the Irish public to do so. Notwithstanding this, there has been an increase in support for recreational use and following a prominent public campaign, Ireland passed legislation allowing for the use of cannabis-based products for a very restricted number of medical purposes. It has been argued that the passage and implementation of legislation to permit the medical use of cannabis has smoothed the transition to recreational cannabis legalization in the USA,^29^ and it remains to be seen whether this will be the case in Ireland. The legalization of recreational cannabis use in the USA and Canada has been described as a large-scale public health experiment, the outcomes of which may be unclear for some time. It has been speculated that the potential effects of legalizing recreational cannabis use include substantially reducing the price of cannabis, increasing heavy use and cannabis-related harms and increasing the number of new users. While it is still too soon to comprehensively assess the impact of cannabis legalization in North America, an understanding of its public health implications will provide important guidance for other countries considering similar legislative changes.^3^

This research has several strengths. This is the first Irish study to examine trends in cannabis attitudes over time and to identify characteristics associated with supporting recreational cannabis use. A further strength of this research is the large sample size and the selection of respondents using random probability sampling that was representative of the Irish population; thus, our findings are generalizable to the whole population. The same methodology was employed for each survey, ensuring that changes in cannabis attitudes over time are not attributable to changes in study design. Despite these strengths, several limitations should be noted. No definition of recreational cannabis was provided to respondents so it is possible that public understanding of this concept may have changed over time, which may have had some impact on our results. In addition, agreement that recreational cannabis use should be permitted does not necessarily imply support for a legal and regulated system of production and supply. Finally, while this study captures trends regarding cannabis attitudes, the survey questions did not allow for exploration of the reasons why respondents hold certain views in relation to cannabis use.

In conclusion, while there has been majority opposition to permitting recreational cannabis use in Ireland since 2002, support has increased in the context of prominent and well-funded campaigns in recent years. When controlling for past use, young adults were found to be most opposed to policy liberalization. Given the potential public health impact of legalization, it is imperative that valid and reliable information on cannabis use, cannabis use disorders and cannabis-related harm is collected, so that the impact of any changes arising from cannabis legalization can be accurately measured.

## Data Availability

The data used and analyzed for the purpose of this study are available from the corresponding author on reasonable request. Key points There is minority support for permitting recreational cannabis use in Ireland; support increased from 19.1% in 2006/07 to 29.9% in 2019/20. Being male, living in Dublin, being either a recent or past cannabis user, knowing cannabis users, perceiving cannabis use as not being a great risk, and not disapproving of cannabis use were significant predictors of agreeing with recreational cannabis use. There was a relative lack of support for recreational cannabis among younger respondents even though younger people in Ireland are more likely to use cannabis compared with older adults. Given the potential public health implications of cannabis legalization, it is imperative that valid and reliable information on cannabis use and cannabis-related harm is collected to ensure that the impact of any changes arising from cannabis legalization can be accurately measured.
